# Determining the feasibility of characterising cellular senescence in human skeletal muscle and exploring associations with muscle morphology and physical function at different ages: findings from the MASS_Lifecourse Study

**DOI:** 10.1007/s11357-023-00869-4

**Published:** 2023-07-11

**Authors:** Leena Habiballa, Adam Hruby, Antoneta Granic, Richard M. Dodds, Susan J. Hillman, Diana Jurk, João F. Passos, Avan A. Sayer

**Affiliations:** 1 AGE Research Group, Faculty of Medical Sciences, Translational and Clinical Research Institute, Newcastle University, Newcastle Upon Tyne, UK; 2 NIHR Newcastle Biomedical Research Centre, Newcastle University and Newcastle Upon Tyne Hospitals NHS Foundation Trust, Newcastle Upon Tyne, UK; 3 Social Genetic and Developmental Psychiatry Centre, Institute of Psychiatry Psychology and Neuroscience, King’s College London, London, UK; 4 Robert and Arlene Kogod Center On Aging, Mayo Clinic, Rochester, MN USA; 5 Department of Physiology and Biomedical Engineering, Mayo Clinic, Rochester, MN USA; 6 University of Southern California, Los Angeles, CA USA

**Keywords:** Human skeletal muscle ageing, Cellular senescence, Muscle morphology, Physical function, Sarcopenia

## Abstract

Cellular senescence may be associated with morphological changes in skeletal muscle and changes in physical function with age although there have been few human studies. We aimed to determine the feasibility of characterising cellular senescence in skeletal muscle and explored sex-specific associations between markers of cellular senescence, muscle morphology, and physical function in participants from the MASS_Lifecourse Study. Senescence markers (*p16*, TAF (Telomere-Associated DNA Damage Foci), HMGB1 (High Mobility Group Box 1), and Lamin B1) and morphological characteristics (fibre size, number, fibrosis, and centrally nucleated fibres) were assessed in muscle biopsies from 40 men and women (age range 47–84) using spatially-resolved methods (immunohistochemistry, immunofluorescence, and RNA and fluorescence in situ hybridisation). The associations between senescence, morphology, and physical function (muscle strength, mass, and physical performance) at different ages were explored. We found that most senescence markers and morphological characteristics were weakly associated with age in men but more strongly, although non-significantly, associated with age in women. Associations between senescence markers, morphology, and physical function were also stronger in women for HMGB1 and grip strength (*r* = 0.52); TAF, BMI, and muscle mass (*r* > 0.4); Lamin B1 and fibrosis (*r* =  − 0.5); fibre size and muscle mass (*r* ≥ 0.4); and gait speed (*r* =  − 0.5). However, these associations were non-significant. In conclusion, we have demonstrated that it is feasible to characterise cellular senescence in human skeletal muscle and to explore associations with morphology and physical function in women and men of different ages. The findings require replication in larger studies.

**Supplementary Information:**

The online version contains supplementary material available at 10.1007/s11357-023-00869-4.

## Introduction

As the most abundant tissue in the human body [1], skeletal muscle undergoes dramatic changes with age [1–3] and exerts a profound effect on human health [3] and survival [4]. Loss of skeletal muscle strength (also known as probable sarcopenia) is a common feature of ageing and is strongly associated with a decline in physical function, poor quality of life, loss of independence, and high healthcare costs [5–9]. Currently, no pharmacological treatments exist to ameliorate the changes in skeletal muscle with age and associated decline in physical function, and although resistance exercise training (RET) appears to be effective, the hypertrophic response is variable and blunted in older compared to younger adults, and RET is not possible for everyone [10]. Better understanding of the underlying biological mechanisms driving skeletal muscle ageing has the potential to inform the development of preventative and therapeutic strategies. To this end, the utilisation of deeply phenotyped cohorts [11] and a lifecourse approach to understanding skeletal muscle ageing [12] holds unique value for advancing translational research [13].

Cellular senescence, one of the hallmarks of ageing [14], is defined as a cell fate in response to various stressors, including telomere dysfunction, oncogenic stimulation, and oxidative stress [15–17]. Senescent cells are characterised by irreversible cell-cycle arrest and the secretion of a range of pro-inflammatory cytokines, chemokines, growth factors, and extracellular matrix degrading proteins termed the senescence-associated secretory phenotype (SASP) [17, 18] that contribute to tissue dysfunction [19]. Senescent cells accumulate with age in multiple human tissues [20] and have been implicated in the pathogenesis of several age-related diseases [20–22]. Importantly, the clearance of senescent cells has been shown to alleviate the ageing phenotype and several age-related pathologies in animal models [23, 24]. Cellular senescence is accompanied by various morphological and molecular changes, including organelle dysfunction, change in nuclear structure, and chromatin remodelling leading to changes in gene expression [17]. Thus, senescent cell phenotypes are heterogenous and currently require multiple core senescence markers for the examination of the senescence burden in the absence of a single universal marker [16, 17, 25].

To date, the senescence profile of ageing human skeletal muscle and its potential functional implications have not been adequately described. Current reports showing increases in the mRNA expression of the senescence-associated genes *p21*, *p16*, and several SASP factors [26–29] fail to determine the spatial distribution of the signal across different cell-types, except in one recent study [30]. Those showing increasing DNA damage in human muscle with age do not distinguish between transient and permanent damage, the latter being an important driver of senescence [30, 31]. A few studies exploring the associations between senescence markers and muscle function in older adults (aged ≥ 60 years) have relied on systemic assessments of the senescence burden in blood plasma and white blood cells but not within the muscle niche [33–35]. None have included middle-aged adults to consider the transition from relative stability to progressive decline in muscle function [36] in relation to cellular senescence.

To fill these gaps, this study used spatially resolved methods (i.e., immunohistochemistry (IHC), immunofluorescence (IF), and RNA and fluorescence in situ hybridisation (RNA-ISH and FISH)) to assess a range of senescence markers in vivo with the following aims:

1. To determine the feasibility of in-depth characterisation of cellular senescence and morphology in human skeletal muscle at different ages
2. To explore sex-specific associations between cellular senescence, skeletal muscle morphology, and physical function in participants of different ages from the MASS_Lifecourse Study.

## Materials and methods

### Study participants

Forty community-dwelling white British men and women aged 45 to 85 years were selected at random from those recruited to the Muscle Ageing Sarcopenia Lifecourse (MASS_Lifecourse) Study as described in the study protocol [11]. Briefly, participants were recruited from north-east England general practises within the North East and North Cumbria Clinical Research Network, clinics within the Newcastle upon Tyne Hospitals NHS Foundation Trust, and the NIHR Bioresource Centre Newcastle. The recruitment started in October 2018 and is ongoing, and it was suspended during the Covid-19 pandemic (March–September 2020). Participants were excluded if taking anticoagulant, antiplatelet, immunosuppressant, or diabetes mellitus medication. Those with a cardiac pacemaker or any other metallic or programmable device and pregnant women were also excluded.

#### Ethics

The study was approved in the UK by the Tyne & Wear South Research Ethics Committee (15/NE/0382) and registered under Clinical Trial #NCT04239495. All participants provided written informed consent.

### Participant characteristics

Table 1 lists participants characteristics and corresponding variable categories including (1) socio-demographic variables (age in years, full-time education, occupational class based on the UK National Statistics Socio-Economic Classification (NS-SEC) from the Office for National Statistics 2021 [37]); (2) health and wellbeing (number of long-term conditions, Short Form 36 questionnaire (SF-36) general health and physical functioning subscales); (3) physical function (maximum grip strength (kg), 5-chair stands (s), gait speed (s/m), SARC-F questionnaire score [38], and EWGSOP2 (European Working Group for Sarcopenia in Older People 2) category (no sarcopenia, probable sarcopenia (low grip strength: < 16 kg in women, < 27 kg in men)) [8]); (4) body composition (appendicular lean body mass (ALM), ALM index (ALMI), body mass index (BMI)); and (5) lifestyle variables (smoking status; daily moderate to vigorous physical activity (MVPA) (minutes)) [S1]. Relevant muscle-related outcomes are described in detail below and other characteristics in Supplementary Information, Appendix 1.

**Table 1:** Characteristics of study participants

Characteristics^a^	Men	Women	*P* value^b^
*n* (%)	24 (60)	16 (40)	
*Socio-demographic*			
Age, years	69.09 (60.51, 74.91)	58.26 (51.59, 63.28)	0.01
Full time higher education			0.83
No	16 (66.7)	12 (75)	
Yes	8 (33.3)	4 (25)	
Occupational class^c^			0.35
Routine/manual	6 (25)	3 (18.75)	
Intermediate	3 (12.5)	5 (31.25)	
Managerial/administrative	15 (62.5)	8 (50)	
*Health and wellbeing*			
Long-term conditions	2 (1, 3)	2 (1, 2.2)	0.99
SF-36: general health	59 (52, 70)	70 (64.5, 75)	0.08
SF-36: physical function	100 (100, 100)	94.44 (89.39, 100)	0.02
*Skeletal muscle health and function*			
Maximum grip strength (kg)	42 (37.5, 45.25)	26 (24.21, 32)	< 0.01
Time for 5-chair stands (s)	10.98 (9.49, 12.91)	10.78 (8.24, 12.31)^d^	0.66
Gait speed (m/s)	1.27 (1.15, 1.32)	1.11 (0.96, 1.23)	0.02
SARC-F, 0	22 (91.67)	14 (87.5)	1
SARC-F, ≥ 1	2 (8.33)	2 (12.5)	
EWGSOP2 category^e^			0.33
None	23 (95.83)	13 (81.25)	
Probable sarcopenia	1 (4.17)	3 (18.75)	
*Body composition*			
Appendicular lean mass (ALM; kg)	25.17 (23.02, 27.18)	16.31 (14.88, 19.41)	< 0.01
ALM index (kg/m^2^)	8.0 (7.49, 8.88)	6.31 (5.83, 7.11)	< 0.01
BMI (kg/m^2^)	26.55 (24.56, 28.24)	27.02 (24.88, 28.29)	0.97
BMI category			0.87
Normal, < 25	7 (29.2)	4 (25)	
Overweight, 25–30	13 (54.2)	10 (62.5)	
Obese, > 30	4 (16.7)	2 (12.5)	
*Lifestyle*			
Smoking			0.46
Never	14 (58.3)	9 (56.2)	
Previous	10 (41.7)	6 (37.5)	
Current	0(0)	1(6.25)	
Daily MVPA (minutes)	23.23 (8.64, 38.51)	21.9 (16.65, 38.25)	0.82

*EWGSOP2* European Working Group for Sarcopenia in Older People 2 (revised definition), *MVPA* moderate to vigorous physical activity, *SARC-F* SARC-F questionnaire, *SF-36* Short Form 36-item questionnaire

^a^Values are *n* (%) or median (interquartile range (IQR))

^b^Chi-squared tests for categorical variables and Wilcoxon rank-sum tests for continuous variables

^c^Highest ranking occupation in household

^d^*n* = 15

^e^No participants classified as confirmed or severe sarcopenia

### Physical function

#### Grip strength

Grip strength (kg) was used as a measure of muscle strength and assessed with a Jamar handheld 5030J1 hydraulic dynamometer (Promedics, UK) following a standard protocol [39]. Each hand was assessed three times; the maximum value of six trials was used in analyses.

#### Chair stands

Participants performed a chair-stand test, measuring the length of time taken to stand up five times from a seated position with the arms across the chest [40].

#### Gait speed

Physical performance was assessed by measuring normal gait speed (m/s) over 4 m [11].

### Body composition

Dual-energy X-Ray absorptiometry (DEXA; Lunar iDXA, GE Healthcare, USA) was used to measure ALM (kg) once and to calculate ALMI (appendicular lean muscle mass (kg) divided by the height-squared in metres; kg/m^2^) and BMI (body weight (kg) divided by the height-squared in metres; kg/m^2^).

### Skeletal muscle biopsy

Skeletal muscle biopsies were collected from the *vastus lateralis* muscle under local anaesthesia from eligible participants using a Weil Blakesley conchotome, embedded in Optimal Cutting Temperature (OCT) compound (Sakura Finetek, Torrance, CA, USA), snap frozen in isopentane cooled in liquid nitrogen. Transverse 10 μm thick cryosections were prepared from OCT blocks (Optimal Cutting Temperature compound; Sakura Finetek, Torrance, CA, USA) on a Bright OTF 5000 cryostat at the Wellcome Centre for Mitochondrial Research laboratory (http://www.newcastle-mitochondria.com/), Newcastle University according to established protocol. The sections were mounted onto SuperFrost Plus slides (Thermo Fisher Scientific, Waltham, MA, USA) and stored at − 80 °C until further processing. Biopsies were not taken from participants judged by a clinician (RMD) not to be feasible (e.g., superficial veins at the biopsy site) [11].

### Cellular senescence markers and assays

Table 2 lists selected markers of cellular senescence used in the study to determine the feasibility of characterising senescence burden in myofibres. These include the percentage (%) of the following: (1) ≥ 2 *p16* positive nuclei, fibres or any ≥ 2 *p16*-positive foci (nuclei and fibres) [S2, S3]; (2) nuclei with ≥ 2 or ≥ 3 signals for breaks at telomeres or TAF (Telomere-Associated DNA Damage Foci) [S4, S5]; (3) γH2A.X-positive nuclei (a DNA damage response protein) as a proportion of TAF-positive nuclei [S6]; and (4) HMGB1 (High Mobility Group Box 1) [S7] and Lamin B1-positive nuclei [S8]. Increase in *p16*- and TAF-positive foci and the loss of HMGB1 and Lamin B1 signals indicate the presence of cellular senescence. Details about senescence markers and corresponding variables used in the associations with muscle-related outcomes in main and supplementary analyses are presented in Suppl. Table S1 in Supplementary Information.

**Table 2:** Markers of cellular senescence and morphological characteristics of skeletal muscle in men and women in the MASS_Lifecourse Study

		Men	Women	*P* value^a^
*Cellular senescence markers*^*b*^	*Abbreviation*			
Percentage of nuclear foci positive for ≥ 2 p16 (%)	%2 + p16Nuclei	4.6 (2.3, 8.5)	5.67 (1.47, 10.74)	0.79
Percentage of fibre foci positive for ≥ 2 p16 (%)	%2 + p16Fibre	2.35 (1.0, 6.8)	0.51 (0, 1.6)	0.02
Percentage of any foci positive for ≥ 2 p16 (%)	%2 + p16Any	12.6 (8.3, 16)	8.56 (5.9, 14.9)	0.36
Percentage nuclei positive for ≥ 2 TAF positive (%)	%TAF2 +	5 (1.8, 7.3)	4 (0, 7.5)	0.48
Percentage nuclei positive for ≥ 3 TAF positive (%)	%TAF3 +	0 (0, 1)	1 (0, 1)	0.56
Percentage of nuclei positive for HMGB1 (%)	%HMGB1 +	27.5 (12.8, 37.8)	25.5 (13.3, 30)	0.53
Percentage of nuclei positive for Lamin B1 (%)	%LaminB1 +	24.5 (19.8, 33.3)	23.5 (20, 27)	0.35
γH2A.X positive as a proportion of TAF positive (%)	TAF% γH2A.X	33.1 (18.8, 43.5)	39.7 (22.3, 44.2)	0.64
*Morphological characteristics*^*b*^				
Minimum Feret (mean A.U.)	Minimum Feret	56.14 (50.14, 63.57)	45.61 (39.19, 53.13)	0.01
Fibre number	Fibre Number	99 (80.5, 111)	140 (100.5, 212)	0.02
Fibrotic area (%)	Fibrotic Area	12.85 (10.09, 17.54)	14.26 (10.55, 16.21)	0.85
Centrally nucleated fibres (%)	CNFs	0.33 (0.0033, 0.83)	0.33 (0.0017, 0.83)	0.78

^a^Differences between men and women analysed using Wilcoxon rank-sum tests

^b^Values shown are median (interquartile range (IQR)) of within participant value

#### Immunofluorescence staining and quantification

Frozen muscle cryosections were first left to air dry for 1 h before fixation in cold 4% PFA in PBS (Santa-Cruz Biotechnology) for 7 min, followed by three 5-min TBST washes. After drawing a hydrophobic barrier with the ImmEdge pen (Vector Laboratories), sections were permeabilised in a methanol gradient: 10 min in 70% methanol, 10 min in 95% methanol, 20 min in 100% methanol, and 10 min in 95% methanol followed by 10 min in 70% methanol, and then washed in TBST three times for 5 min each. Next, sections were blocked in normal goat serum (Vector Laboratories) (10%) in TBST at room temperature for 1 h and incubated in Wheat Germ Agglutinin (WGA) for 20 min at room temperature. After three 5-min washes with TBST, sections were incubated with primary antibody overnight at 4 °C. Following another three 5-min TBST washes, sections were incubated with secondary antibody for 2 h in a humidified chamber at 4 °C. Slides were then mounted using ProLong Gold Antifade Mountant with DAPI (Invitrogen).

WGA was used to delineate myonuclei from interstitial and satellite cells. For HMGB1, a nuclear mask of the DAPI channel was created for each image, and the mean intranuclear fluorescence intensity of each nucleus was measured in the relevant channel of each image. Nuclei were classified as positive if their mean nuclear fluorescence intensity was higher than the total sum of the sarcomeric fluorescence intensity and twice the standard deviation (SD) of the sarcomeric fluorescence intensity for the respective channel in the respective image from which each nucleus was measured. 100–250 nuclei were analysed per sample. For Lamin B1, an eroded nuclear mask was created using the DAPI channel of each image and subtracted from the original Lamin B1 channel, leaving only the peripheral area of the nuclei constituting the Lamin B1 ring. The integrated nuclear density of the Lamin rin’s final mask was calculated in the Lamin B1 channel for 100–250 nuclei per sample. Touching nuclei or nuclei that displayed a blurred DAPI signal were not analysed.

#### ImmunoFISH staining and quantification

For frozen muscle cryosections, immunohistochemistry was carried out as described above. After the addition of WGA, sections were rinsed with TBST and incubated in avidin for 15 min, followed by a PBS rinse and an incubation in Avidin/Biotin Blocking Kit (Vector Laboratories) for 15 min at room temperature. Sections were rinsed once more and incubated with primary antibody overnight at 4 °C. Following an overnight incubation with rabbit monoclonal anti-γH2A.X (Cell Signaling, Danvers, MA, USA), sections were then incubated with a goat anti-rabbit biotinylated secondary antibody (Vector Laboratories) for 1 h at room temperature. Following three 5-min TBST washes, tissues were incubated with fluorescein avidin DCS (Vector Laboratories) for another 20 min at room temperature. Sections were then washed three times in PBS and cross-linked by incubation in 4% PFA (Sigma) in PBS for 20 min. Sections were washed in TBST three times and then dehydrated in graded cold ethanol solutions (70, 90, and 100%) for 3 min each. Tissues were then allowed to air dry prior to being denatured in 10 µl of PNA hybridization mix (70% deionised formamide (Sigma), 25 mM MgCl_2_, 1 M Tris pH 7.2, 5% blocking reagent (Roche) containing 2.5 μg/ml Cy-3-labelled telomere-specific (CCCTAA) peptide nucleic acid probe (PANAGENE, Yuseong-gu, Daejeon, South Korea)) for 10 min at 80 °C and then incubated overnight at 4 °C in a dark humidified chamber to allow hybridisation to occur. The following day, sections were washed in 70% formamide in 2 × SCC for 10 min, followed by a wash in 2 × SSC for 10 min, and a PBS wash for 10 min. Tissues were then mounted using ProLong Gold Antifade Mountant with DAPI (Invitrogen). Sections were imaged for TAF using in-depth Z stacking (a minimum of 40 optical slices with 63 × objective) followed by Huygens (SVI) deconvolution and ImageJ analysis. WGA was used to delineate myonuclei from interstitial and satellite cells. 100 myonuclei were analysed per sample.

#### RNA in situ hybridisation (RNA-ISH) and quantification

RNA in situ hybridisation was performed using the RNAscope 2.5 HD Detection Reagent–RED Assay (Advanced Cell Diagnostics, Newark, CA, USA) following the manufacturer’s instructions. Briefly, sections were removed from − 80 °C and fixed in 4% PFA for 20 min, washed in PBS, and dehydrated with increasing concentrations of ethanol (50%, 70%, and 100%). After drawing a hydrophobic barrier with the ImmEdge pen (Vector Laboratories), sections were incubated with hydrogen peroxide for 10 min at room temperature, washed with distilled water, and incubated with Protease IV for 15 min at room temperature. Sections were then washed in PBS and incubated for 2 h at 40 °C with the RNAscope probe targeting *p16* (Advanced Cell Diagnostics). After rinsing with wash buffer, sections were incubated at 40 °C with Amp 1 (30 min), Amp 2 (15 min), Amp 3 (30 min), and Amp 4 (15 min) and incubated at room temperature with Amp 5 (30 min) and Amp 6 (15 min) with rinses in wash buffer at room temperature between each incubation step. Sections were next incubated at room temperature with 1:60 RED-B to RED-A solution for 10 min. After a wash in distilled water, sections were incubated for 2.5 min in 50% Haematoxylin and then washed three times in distilled water. Sections were dried for 5 min at 60 °C, briefly washed in Histo-Clear (National Diagnostics) and mounted with EcoMount (Biocare Medical). Sections were imaged using light microscopy at 20X. Image analysis was carried out using the open-source Fiji software [41]. The number of p16 foci in each myofibre was counted, including its localisation within or outside a nucleus. 75 to 125 myofibres were analysed for each section.

Suppl. Tables S2 to Suppl. Table S4 in Supplementary Information list reagents, probes, solutions, antibodies, and their dilutions used in the protocols described above.

For *p16* expression, 75 to 125 myofibres were examined for each muscle section as described above. For TAF evaluation, at least 100 myonuclei were analysed, and for quantification of HMGB1 and Lamin B1, 100–250 nuclei per biopsy were examined. Muscle sections were imaged using either in-depth Z-stacking followed by ImageJ analysis (for TAF) or with the open-source image processing package, Fiji (https://imagej.net/software/fiji/) of ImageJ2 (for *p16*) [41].

### Morphological characteristics and assays

Table 2 lists selected morphological characteristics used in the study: the minimum Feret diameter (arbitrary units; A.U. for fibre size), fibre number, proportion of fibrotic area (%), and centrally nucleated fibres (CNFs, %). Corresponding immunohistochemistry assays for the parameters assessment, such as the haematoxylin and eosin (H&E) staining and picrosirius red (PR) staining, and the procedure for parameter measurement are described in detail in below.

#### Haematoxylin and Eosin (H&E)

Frozen cryosections were left to air dry for 1 h before fixation in cold 4% PFA in PBS (Santa-Cruz Biotechnology) for 7 min, followed by three 5-min TBST washes. Sections were stained in Haematoxylin (Sigma-Aldrich) for 1 min, then washed three times in distilled water for 1 min per-wash followed by further washing in warm running tap water for 20 s on the reverse side of the slides. Sections were then washed in distilled water once for a 1 min. Eosin-Y (ScyTek Laboratories) was used to counterstain sections for 15 s followed by a wash under running tap water on the reverse side of the slides to remove residual stain. Once clear, sections were then dehydrated in 95% and 100% ethanol twice each for 30 s, then washed with 100% Histoclear twice for 5 min. Next, sections were mounted using EcoMount and allowed to set. Slides were imaged at 20X magnification and analysed using ImageJ. For each slide, 20–30 images were taken randomly across the whole length of the section, ensuring equal distribution of fibres and a total number of 300–500 fibres imaged per sample (biopsy). Using the freehand select tool on ImageJ, 300 fibres per sample were manually outlined and the minimum feret diameter and cross-sectional area for each recorded. The number of fibres within a square millimetre of tissue and the number of centrally nucleated fibres were also manually recorded.

#### Picrosirius Red (PR)

Frozen sections were left to air dry for 1 h before fixation in cold 4% paraformaldehyde (PFA), followed by hydration in distilled water. Sections were incubated in the Picrosirius Red solution (Picrosirius Red Stain Kit; ab150681; Abcam) for 60 min at room temperature and rinsed twice in acetic acid solution. Sections were then rinsed in absolute alcohol and dehydrated by two quick changes in absolute alcohol and finally mounted in DPX (Dibutylphthalate Polystyrene Xylene) mounting media for microscopy. For fibrosis analysis, a tile scan of sections was performed and stitched post-imaging. Images were inverted on ImageJ, and using a threshold, the area fraction occupied by fibrotic red material was measured for each section.

For H&E assay, 20–30 images were taken randomly across the entire length of the section per slide to ensure equal distribution of myofibres and 300–500 fibres imaged per biopsy. Of those, at least 300 fibres were manually outlined using ImageJ software (https://imagej.nih.gov/ij/index.html; National Institute for Health) and the minimum Feret diameter, number of fibres (per mm^2^ of tissue), and percentage of CNFs recorded. For PR assay (fibrosis analysis), a tile scan of sections was stitched after imaging, and images inverted with ImageJ. Using a threshold, the area fraction occupied by fibrotic red staining was measured for each section.

Suppl. Table S2 and Suppl. Table S3 list reagents and stock solutions used in H&E and PR assays.

### Microscopy

For all immunofluorescence images, fluorescence microscopy was conducted with a Leica DM5500B widefield fluorescence microscope and Leica DMI8 (Leica Microsystems Inc., Deerfield, IL. USA). For immunohistochemistry images, light microscopy was conducted using a Nikon Eclipse E-800 Brightfield camera (Nikon Metrology Inc., Brighton, MI, USA). All image acquisitions were performed with the Leica LASX software.

### Statistics

GraphPad Prism 9 (GraphPad Software, San Diego, CA, USA) and R version 4.0.2 (R Foundation for Statistical Computing, Vienna, Austria; https://www.R-project.org/) were used for the analyses. Data quantification for senescence markers and morphological characteristics was performed blinded and expressed as either percentages (e.g., the proportion of ≥ 2 TAF-positive nuclei), counts (e.g., fibre number), or means (e.g., fibre size). Men and women were described using descriptive statistics across key socio-demographic, physical function, body composition and lifestyle variables, and their differences analysed using a *χ*^2^ test for categorical variables (counts and percentages) and Wilcoxon rank sum tests for continuous variables (medians with interquartile range, IQR) at *p* < 0.05 (Table 1). Correlations (i.e., associations between senescence markers, morphological characteristics, and muscle-related outcomes in men and women with age) were explored using Spearman’s rank correlation test to examine how the pairs of variables of interest change together and not necessarily at a constant rate. Statistical power was calculated in MATLAB® version R2021a (TheMathWorks Inc., Natick, MA, USA). Because of small sample size (< 50), low power, and significant number of correlations examined, their statistical significance evaluated at α < 0.01 was not reported. Thus, only correlation coefficients were presented to highlight the direction and strength of the associations. To interpret the magnitude of *r*, Cohen’s (1988) benchmarks for *r* = 0.1 as small, 0.3 as medium, and 0.5 and over as large effect size were used [42].

**Fig. 1:**
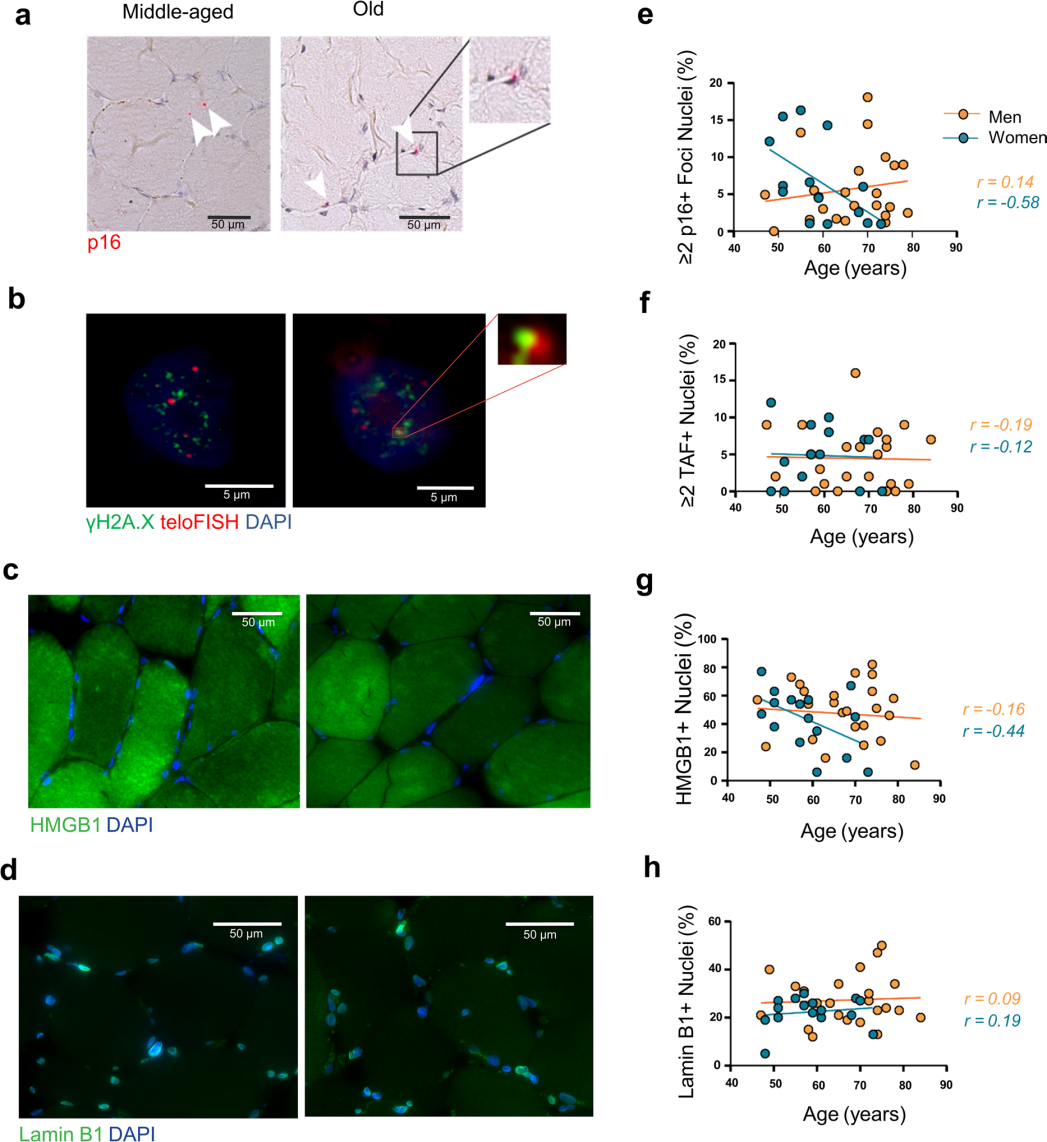
Expression of markers of cellular senescence in myonuclei of skeletal muscle with age in men and women in the MASS_Lifecourse Study. Representative images portraying **a** p16 (red) RNA in situ hybridisation staining, **b** ImmunoFISH- telomeres (red), γH2A.X foci (green), and **c** HMGB1 (green) and **d** Lamin B1 (green) immunofluorescence staining. White arrows indicate p16-positive nuclei or areas of co-localisation between telomeres and γH2A.X foci. Graphs of correlations between age and the percentage of **e** p16-postitive nuclei, **f** TAF-positive myonuclei, **g** HMGB1-positive myonuclei, and **h** Lamin B1-positive myonuclei in middle-aged and old men (orange circles) and women (teal circles) in the MASS_Lifecourse Study. Correlations were examined using Spearman’s correlation test. Regression lines for men represented in orange, and in teal for women. Graphs were generated in Prism 9.0

**Fig. 2:**
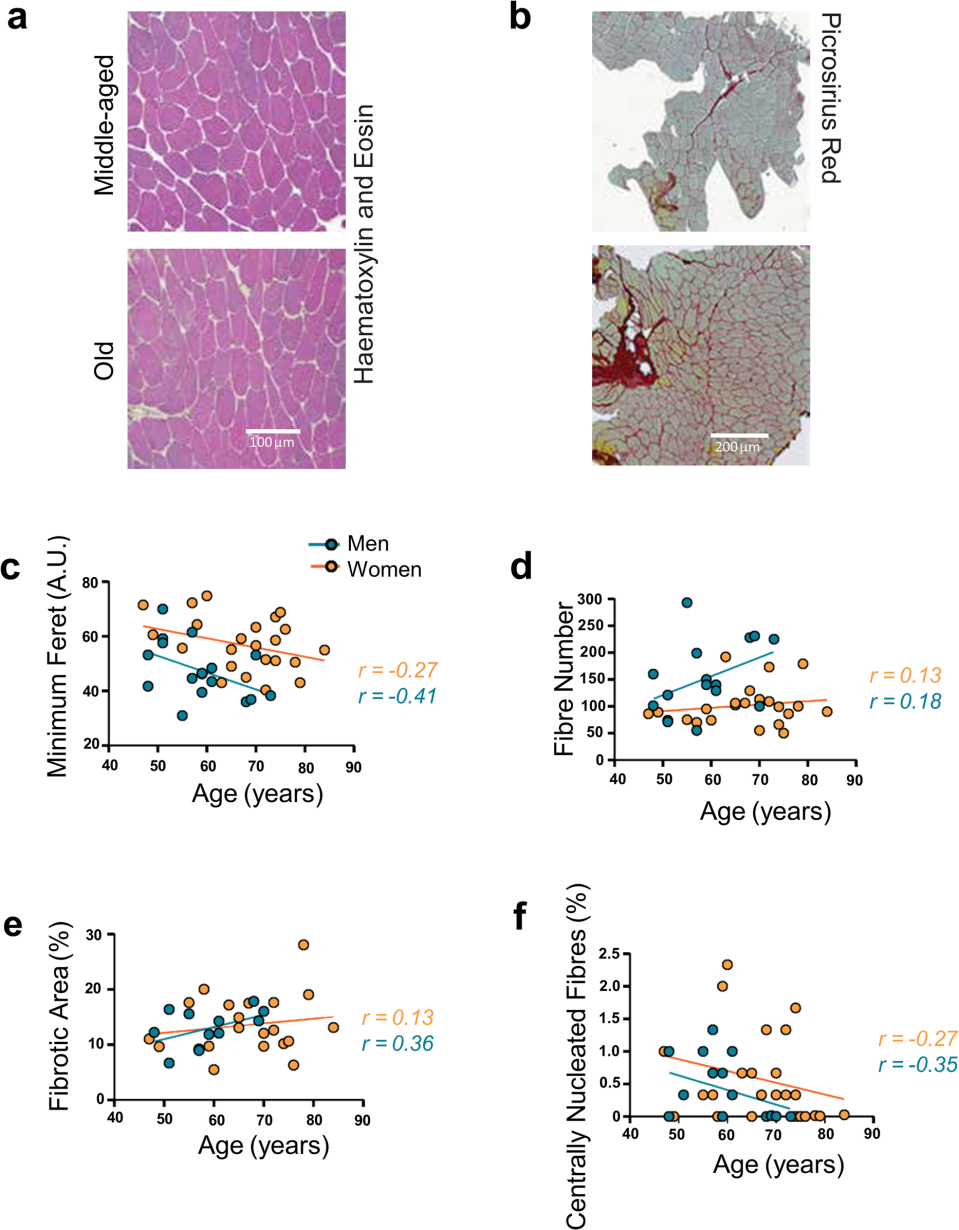
Morphological characterisation of skeletal muscle with age in men and women in the MASS_Lifecourse Study. Representative images of **a** haematoxylin and eosin staining and **b** picrosirius red staining in middle-aged and old skeletal muscle. Graphs showing correlations between age and **c** mean minimum Feret, **d** fibre number, **e** the percentage fibrotic area, and **f** the percentage of centrally nucleated fibres in men (orange circles) and women (teal circles). Correlations were examined using Spearman’s correlation tests. Regression lines for men represented in orange, and in teal for women. Graphs were generated in Prism 9.0

**Fig. 3:**
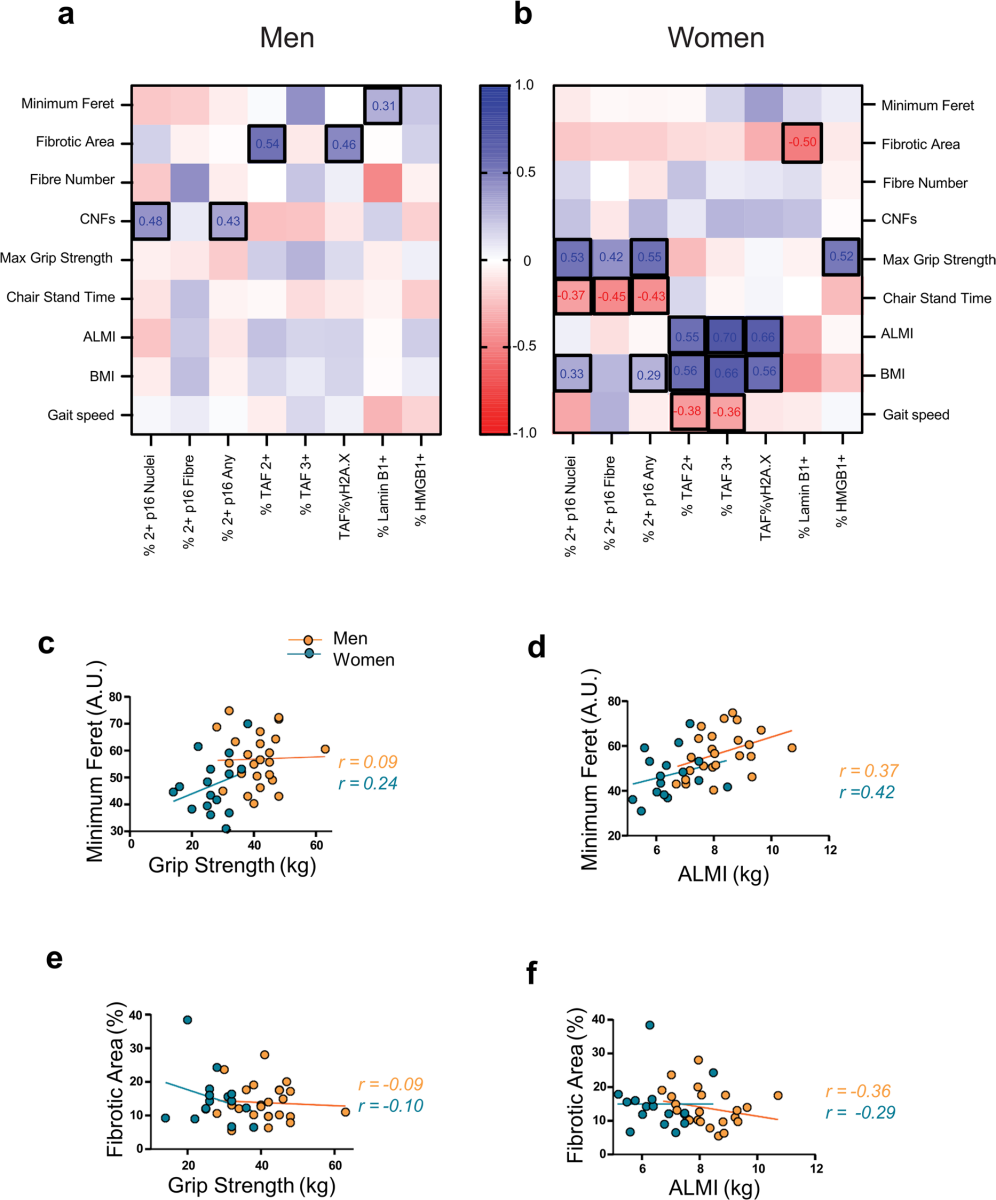
Associations between cellular senescence, morphological parameters, and skeletal muscle health and function with age in men and women in the MASS_Lifecourse Study. Heatmaps portraying correlation coefficients between senescence markers, physical function, and indicators of muscle ageing in middle-aged and old **a** men and **b** women in the MASS_Lifecourse Study. Graphs showing correlations between grip strength and **c** fibre size, **e** percentage fibrotic area, as well as between appendicular lean mass index and **d** fibre size, and **f** percentage fibrotic area in men (orange circles) and women (teal circles). Regression lines for men represented in orange, and in teal in women. Heatmaps and graphs were generated in Prism 9.0

In the main analyses, the following associations were explored in men and women: (1) cellular senescence markers and age; (2) associations between morphological characteristics and age, and (3) senescence markers, morphological characteristics, and physical function/body composition (Figs. 1, 2, and 3). Additional associations were explored in supplementary analyses (Suppl. Fig. S1 to Suppl. Fig. S3 in Supplementary Information).

## Results

### Participant characteristics

Forty participants (age range 47.3–84.2 years, 40% women) recruited as part of the MASS_Lifecourse Study underwent deep phenotyping and functional assessments, including for physical function. Men exhibited higher average age, grip strength, ALM and ALMI, SF-36 physical function score, and faster gait speed compared with women. No other differences between men and women were observed across several key characteristics (Table 1). Only four participants (three women) had probable sarcopenia (low grip strength), indicating a healthy cohort. As expected, significant correlations were observed between measures of muscle strength and mass (grip strength and ALM) with age in men and women (*r* ≥  − 0.5; Appendix 2, Supplementary Information), indicating decline in physical function with ageing. No other changes were observed with age in either gender (e.g., gait speed, BMI, ALMI, and MVPA) (Appendix 2, Supplementary Information).

### Characterisation of cellular senescence burden in skeletal muscle in men and women with age

Figure 1a–h depicts change in cellular senescence markers in skeletal muscle biopsies with age. Figure 1a–d shows representative images of four core cellular senescence markers that were used in this study to investigate the senescence burden in skeletal muscle in the absence of a stand-alone marker. Figure 1a shows an RNA-ISH staining representing the expression of the cyclin dependent kinase inhibitor (CDKI) *p16* in middle-aged and older muscle [S2, S3]. In women, there was a strong negative correlation between the percentage of nuclei (*r* =  − 0.58), but not fibres (*r* =  − 0.27) expressing ≥ 2 *p16* mRNA transcript foci with age, suggestive of decreased *p16* gene expression in myonuclei with ageing. These changes were not observed in men (*r* < 0.20), although men overall had significantly higher percentage of fibres positive for ≥ 2 *p16* foci (*p* = 0.02) compared with women (Table 2, Fig. 1e).

Figure 1b depicts a representative image of telomere dysfunction in middle-age and older myonuclei, visualised by immuno-FISH, showing the colocalisation between telomeres (in red) and the DNA-damage response (DDR) protein γH2A.X (in green), also known as TAF (Telomere-Associated DNA Damage Foci) [S4-S6]. Weak negative correlations were detected between the percentage of nuclei expressing ≥ 2 TAF and age in both men and women (Fig. 1f). No differences between men and women were observed in any TAF or γH2A.X associated markers presented as medians (IQR) (Table 2 and Suppl. Table S5). Women had higher percentage of γH2A.X positive foci (DNA damage) as a proportion of TAF positive nuclei (40% versus 33% in men) which was not statistically significant (Table 2).

Figure 1c, d shows representative images of two cellular senescence markers involved in chromatin remodelling and transcriptional reprogramming during senescence assessed by immunofluorescence: the HMGB1 (High Mobility Group Box 1) alarmin protein (Fig. 1c in green) and Lamin B1 (a nuclear envelope protein) (Fig. 1d in green) [S7, S8]. Intranuclear HMGB1 was negatively associated with age in women (*r* =  − 0.44) (Fig. 1g), and weak positive correlations were observed between Lamin B1 expression and age in both genders (*r* < 0.20) (Fig. 1h), suggesting a loss of HMGB1 in women and no change in Lamin B1 with ageing.

Suppl. Fig. S1 (panels a–f) shows the results of additional correlations between (1) senescence markers and age (panels a, b, d, and e) and (2) the associations between all markers used in the study in men and women (heatmaps, panels c and f, respectively). Moderate to strong negative correlations were observed for the percentage of *p16*-positive nuclei (*r* =  − 0.64) (panel a), *p16*-positive fibres (*r* =  − 0.44) (panel b), and *p16*-positive nuclei and fibres together (*r* =  − 0.71) (panel d) in women, but not in men, with age. In both, the percentage of nuclei expressing ≥ 3 TAF was moderately associated with age (*r* =  − 0.5 in men and − 0.3 in women), indicating loss of 3 + TAF nuclei with ageing (panel e). Heatmaps representing the associations between senescence markers revealed different tile patterns between men and women (e.g., a strong correlation between the percentage of fibres positive for ≥ 2 *p16* foci and γH2A.X-positive nuclei in the upper right quadrant in women, but not in men) (panels c and f).

Taken together, the results indicate the feasibility of cellular senescence burden characterisation using several senescence markers in skeletal muscle in men and women across different ages.

### Morphological characteristics of skeletal muscle biopsies in men and women with age

A comparison of median values of morphological characteristics between men and women returned no significant gender differences except for minimum Feret diameter (fibre size) which were smaller overall in women (Table 2). Changes in skeletal muscle morphological characteristic with age are presented in Fig. 2a–f. Figure 2a, b depicts changes in muscle health and quality with age, such as an increase in fibrosis in old versus middle-aged muscle (Fig. 2b). Correlation analyses between morphological characteristics and age revealed overall stronger effects in women, with decline in fibre size (*r* =  − 0.41) and percentage of centrally nucleated fibres (*r* =  − 0.35), and an increase in percentage of fibrotic area (*r* = 0.36) with age compared with men (*r* =  − 0.27, *r* =  − 0.27, and *r* = 0.13, respectively) (Fig. 2c, e, f). Correlation coefficients for fibre number were small in both men and women (*r* < 0.2), suggesting nonsignificant changes with age (Fig. 2d).

### Associations between cellular senescence and physical function in men and women with age

Further associations explored whether the molecular senescence signature associate with age-related decline in muscle health and function (Fig. 3a, b), and correlations of interest are presented via heatmaps for men and women (tiles with bold black borders). No strong correlations between functional measures and senescence markers were observed in men (Fig. 3a). In women, *p16* expression exhibited moderate to strong negative correlations with 5-chair stand time (*r* >  − 0.4), and positive correlations with BMI and grip strength (*r* > 0.3). Similarly, TAF was negatively correlated with gait speed (*r* =  − 0.36), but positively associated with BMI and ALMI in women with age (*r* > 0.55). Finally, HMGB1 was positively associated with grip strength in women (*r* = 0.52) (Fig. 3b).

Correlations between senescence markers and morphological characteristics displayed strong positive associations between TAF and fibrosis, as well as *p16* and percentage CNFs in men (all *r* > 0.40), but not in women. However, Lamin B1 was negatively associated with fibrosis in women only (*r* =  − 0.50) (panel b). All other correlations not reported in Fig. 3 are presented in the heatmaps in Suppl. Fig. S2 (Appendix 3, Supplementary Information).

### Associations between skeletal muscle morphology and physical function in men and women with age

Further associations explored whether morphological health was an indicator of functional status by correlating the most consistent indicators of morphological health—fibre size and fibrosis—with grip strength and ALMI (Fig. 3c–f). Only weak correlations were observed between grip strength and fibre size as well as fibrotic area in men (*r* = 0.09 and *r* =  − 0.09, respectively), whereas grip strength and fibre size (but not fibrosis) revealed a stronger correlation in women (*r* = 0.24) (Fig. 3c, e). ALMI showed positive associations with fibre size (*r* ≥ 0.36) and negative associations with fibrotic area in men and women (*r* =  − 0.36 and − 0.29, respectively) with ageing (Fig. 3d, f).

Supplementary analyses between these morphological parameters and other measures of muscle health and function (chair stands, gait speed, BMI, and MVPA) showed mostly weak correlations (Suppl. Fig. S3), except for a positive association between gait speed and fibrotic area in men (*r* = 0.3), and a negative association between gait speed and fibre size in women (*r* =  − 0.5) with age (Suppl. Figure 3, panels d and h, respectively).

## Discussion

This study successfully demonstrated that it is feasible to carry out in-depth characterisation of cellular senescence in human skeletal muscle in relation to physical function at different ages using participants from the unique MASS_Lifecourse Study. A range of advanced spatially-resolved techniques was deployed to establish the nature of the senescence phenotype i*n vivo.*

Previous studies assessing the senescent cell burden in human skeletal muscle and its relationship to physical function measured biomarkers systematically in blood rather than locally within the muscle niche and relied on bulk analyses lacking the power to determine the spatial distribution of the senescence signal across different cell-types [26, 33–35], whilst assessments of DNA damage in the muscle could not distinguish between transient and irreversible damage [31, 32]. To our knowledge, this study is the first to demonstrate the feasibility of an in-depth characterisation of senescence *in vivo* in relation to relevant muscle-related outcomes.

As no stand-alone marker can be used to determine the senescent state, this study employed four markers and derived a range of senescence variables (Fig. 1, Table 2, Suppl. Table S1). HMGB1 and Lamin B1 expression were used to assess for chromatin changes associated with the senescent state [S7, S8], whilst telomeric damage and *p16* expression were assessed as indicators of early events in the induction of the senescence programme [S2-S5]. This study revealed no compelling relationships between any of the markers and age. An exception was a tendency towards the loss of HMGB1 in women from mid-life onwards as is typical in senescence, suggesting that perhaps sex-specific changes may be relevant for age-related HMGB1 expression. Our recent study that used spatially resolved techniques to assess skeletal muscle senescence [30] has demonstrated that p16 increased significantly in older human muscle compared to young muscle. Conversely, our findings suggest no change from middle age and overall negative correlation (moderate to strong in the percentage of nuclei, nuclei plus fibre positive for *p16*) in women only, potentially pointing to gender differences and the bulk of the p16 burden possibly accumulating earlier in life. However, given the moderate effect size, low number of samples, and low power of these analyses, the interpretation of the results is limited.

Similar to senescence markers, morphological characterisation of muscle biopsies revealed no strong correlations between morphological characteristics and age but tended towards a more exacerbated ageing phenotype—particularly for fibre size and fibrosis—in women compared with men, but not for fibre number in both genders (Fig. 1a–f). Although these analyses offer a snapshot of mid- to late life changes that contributes to our understanding of muscle changes across the lifecourse, they largely do not reflect the dominant ageing phenotype for skeletal muscle [36, 43–45] and may indicate a healthier cohort. Muscle quality and performance appear to be relatively preserved in this study, suggesting that chronological age may not be a steadfast indicator of muscle health in these individuals, and that additional molecular data and other biological factors should be considered.

Furthermore, exploration of the relationship between senescence markers and physical function with age revealed no striking correlations. The associations in men were weak, and although stronger (effect sizes > 0.4) in women, they were inconsistent and conflicting, particularly for *p16* and TAF. In women, *p16* was negatively associated with 5-chair stands, whilst TAF was positively associated with BMI and muscle mass (*r* > 0.55), but negatively associated with gait speed (*r* =  − 0.4). On the other hand, men exhibited somewhat stronger associations between senescence markers TAF and *p16* and morphological characteristics (fibrosis and CNFs, respectively) compared with women.

Similarly, only few noticeable associations were observed between morphological characteristics and muscle function, which were stronger in women for grip strength and gait speed in relation to fibre size (*r* = 0.24 and *r* =  − 0.5, respectively). Furthermore, muscle mass (ALMI) had moderate positive association with fibre size and negative association with fibrosis in men and women with ageing. Taken together, muscle quality and performance appear to be relatively preserved in this study, suggesting that chronological age may not be a reliable indicator of muscle health in these individuals and that other biological factors may play a greater role.

Given the exploratory nature of the analyses and inconsistency of the results, they should be interpreted with caution when considering the possibility of sex-specific differences in the patterns observed. The inconsistencies could be contributed to low statistical power and biassed sampling. Although assessed in blood samples, a recent study by Fielding et al. (2022) [35] demonstrated compelling relationships between biomarkers of senescence (components of SASP) and measures of muscle strength and function in a cohort of over 1300 older adults aged 70–89 years, suggesting that higher numbers may be required to run sensitive enough analyses of such associations *in vivo*. This study also revealed for the first time that circulating components of the SASP can be effectively assessed as biomarkers of senescent cell burden in a large number of people in relation to various parameters of muscle function [35]. The MASS_Lifecourse participants included in this study had low prevalence of probable sarcopenia, had high SF-36 scores on physical functioning subscale, and were physically active indicating a sample of healthy individuals. The evaluation of the first 80 participants in the MASS_Lifecourse Study [11] also revealed good physical function, low rates of sarcopenia, and grip strength meaning that observations made in this cohort may not represent the general population leading to selection bias.

It is hoped that a better understanding of the nature and mechanisms of cellular senescence will enable better long-term therapeutics in the form of senolytics—drugs that specifically eliminate senescent cells [46]. Recently, human clinical trials of senolytics launched in patients with idiopathic pulmonary fibrosis (IPF), systemic sclerosis, chronic kidney disease, and diabetes reported a decrease in the senescent cell burden and the alleviation of physical dysfunction in treated subjects [47, 48; ClinicalTrials.gov Identifier: NCT02848131]. Although these precedents present a promising prospect for the treatment of muscle ageing and sarcopenia, it should be acknowledged that not all interventions with senolytics have been beneficial for musculoskeletal health in pre-clinical studies with aged rodents [49] and should be approached with caution in human clinical trials [50].

Currently, rodent studies are used as a benchmark for the translational leap in senotherapy despite major difficulties in translating protocols between species and accounting for gender differences in humans. Moreover, recently Börsch and colleagues (2021) showed that the most pronounced changes in cellular senescence and inflammation pathways in skeletal muscle occur relatively earlier in humans (40–49 years) than in mice (26–28 months—equivalent to over 70 in human years) [51]. Despite a sample size of only 40 individuals, our results suggest that shifts in expression of senescence-associated markers do occur in humans during middle-age, and this early onset might imply that anti-ageing interventions should start at this critical period of life that is marked by the start of decline in physical function [36]. Further studies with well-characterised and powered cohorts inclusive of younger participants are needed to determine the ‘therapeutic window’ of such interventions.

In addition to exploring the senescence phenotype in nuclei and muscle fibres, future human studies will also benefit from exploring the senescence phenotype in different muscle, fibres, and other cell types within the muscle niche. Studies show that gene expression profiles of different skeletal muscle types vary [52], and this may extend to their senescence profiles. This is especially relevant given the varying levels of resistance to oxidative stress—one major driver of the senescence response—exhibited by predominantly glycolytic, oxidative, or mixed muscle groups [53]. Powerful techniques such as single cell RNA sequencing [54], MACS (Magnetic Activating Cell Sorting), and Gene Set Enrichment (GSEA)-based approaches (e.g., the SenMayo gene set) [55] will enable the examination of transcriptional changes in various mononuclear cell populations in skeletal muscle during ageing. Single myofibre isolation techniques will also enable better understanding of the distribution of the senescence signature across different myofibre types. It is likely that subsequent studies in the field will use a combination of all these techniques moving forward.

### Strengths and limitations

This study has several strengths, including the first ever determination of the feasibility of characterising a number of cellular senescence markers and morphological characteristics in muscle biopsies of middle-aged and older participants belonging to a deep phenotyped, unique cohort. The senescence burden was described with a range of variables and the strength of correlations with several measures of muscle health and function explored in men and women. However, the study has several limitations that need to be considered when interpreting the results. First, only four senescence markers were used, and the study was unsuccessful to characterise the senescence burden with two other commonly used senescence markers, p21 and SA-β-Gal [30, 56]. Second, morphological characteristics did not include the analyses of fibre typing, their change with age, and gender differences which are planned for the larger MASS_Lifecourse Study [11]. This also implies that the median values of fibre size were based on the minimum Feret diameter of various fibres. Third, the study did not measure SASP [35], but concentrated on the senescence burden within the muscle niche which was deemed to be more relevant for the muscle parameters used in correlations. Fourth, in the absence of a direct measure of quadriceps strength, grip strength was employed as a surrogate measure. Numerous epidemiological cohort studies have established that grip strength correlates well with strength of other muscle groups including lower limb muscles in older adults [57] and associates strongly with relevant health outcomes [58]. Lastly, the study was underpowered to draw any conclusions about the trends observed, and any relevant results have limited generalisability to white British adults.

In summary, using participants from the unique MASS_Lifecourse cohort, this study has demonstrated that it is feasible to characterise cellular senescence in human skeletal muscle and to explore associations with morphology and physical function in women and men of different ages. The observed associations now need replication in larger studies.

### Supplementary Information

Below is the link to the electronic supplementary material.

Supplementary file1 (PDF 849 KB)

## Data Availability

Due to the funder (NIHR) regulations of data usage, data are available upon request from responsible PI.

## Declarations

### Disclaimer

The views expressed are those of the authors and not necessarily those of the National Institute for Health and Care Research, United Kingdom.
