# Supplementary material for: Determining the feasibility of characterising cellular senescence in human skeletal muscle and exploring associations with muscle morphology and physical function at different ages: findings from the MASS_Lifecourse Study
Source: GeroScience. 2023 Jul 11;46(1):1141–58. doi: 10.1007/s11357-023-00869-4 (PMC10828484; doi:10.1007/s11357-023-00869-4)
Supplement: Supplementary file 1 — Supplementary file1 (PDF 849 KB) [file 11357_2023_869_MOESM1_ESM.pdf]

# Supplementary Information

## Determining the feasibility of characterising cellular senescence in human skeletal muscle and exploring associations with muscle morphology and physical function at different ages: findings from the MASS_Lifecourse Study

Corresponding author: Antoneta Granic[1,2]

Email: antoneta.granic@newcastle.ac.uk

[1]AGE Research Group, Translational and Clinical Research Institute, Faculty of Medical Sciences, Newcastle University;

[2] NIHR Newcastle Biomedical Research Centre, Newcastle University and Newcastle Upon Tyne Hospitals NHS Foundation Trust, Newcastle upon Tyne, UK;

# Appendix 1

## Materials and Methods

### Participant characteristics

*Skeletal muscle health and function*

Sarcopenia categories were based on the revised EWGSOP2 definition of sarcopenia (the European Working Group on Sarcopenia in Older People) [9]. Briefly, participants were classified as having probable sarcopenia if having low grip strength (<27 kg in men and <16 kg in women) and/or longer time for 5-chair stands (>15 s); confirmed sarcopenia if additionally having low appendicular lean mass index (ALMI; ALM in kg divided by height$^2$ in m (<7.0 kg/m$^2$ in men and <5.5 kg/m$^2$ in women)), and severe sarcopenia if having also slow gait speed (≤ 0.8 m/s).

*Lifestyle*

*Daily moderate to vigorous physical activity (minutes)*: Participants were provided with a GENEActiv® wrist-worn accelerometer (calibrated to 100Hz) and were instructed to wear it continuously for 7 days on a dominant wrist. This device measures the acceleration of the wearing arm along three different axes of space in milligravity units (mGal) as the arm moves, thus denoting the amount/intensity of physical activity of the wearer. Participants were provided with a form to record any time intervals during which they removed the device. The accelerometers were collected at a clinical visit during which muscle biopsy and imaging were performed. From the GeneActiv

accelerometer recordings, the mean daily duration of moderate to vigorous physical activity (MVPA) was calculated using the GGIR package in R (https://cran.r-project.org/web/packages/GGIR/GGIR.pdf) [S1]. MVPA was defined as time periods for which measured acceleration was >0.1 *g* calculated using the Euclidean norm minus one algorithm, which were sustained for at least 80% of a minimum bout of 5 minutes as described in the MASS_Lifecourse Study protocol [11].

# Appendix 2

## Supplementary Results

### Associations between muscle-related outcomes and age

*Men*

The following significant Spearman correlations were observed in men between measures of muscle strength and mass with age: grip strength ($r$ = -0.53), and ALM ($r$ = -0.48) showing a decline in muscle health with ageing. Associations with gait speed, 5-chair stands, BMI, ALMI, MVPA and 5-chair stands were weak ($r$ = -0.18, $r$ = 0.33, $r$ = -0.24, $r$ = -0.37, and $r$ = -0.38, respectively).

*Women*

Similarly, a significant Spearman association was found between ALM ($r$ = -0.66) and age in women, but not with grip strength ($r$ = -0.49), gait speed ($r$ = -0.08), BMI ($r$ = -0.31), ALMI ($r$ = -44), and MVPA ($r$ = -0.36) compared with men.

## Supplementary Tables

**Supplementary Table 1.** Description of cellular senescence markers and corresponding variables in the MASS_Lifecourse Study

| **Cellular senescence marker** | **Abbreviation** | **Description** | **Method of detection** |
|---|---|---|---|
| *p16* | | The cyclin dependent kinase inhibitor (CDKI) *p16* is a primary mediator of cell-cycle arrest and a promoter of cellular senescence [S2, S3, 21]. | RNA *in situ* hybridisation (RNA-ISH) |
| Percentage of nuclear foci positive for ≥2 *p16* (%) | %2+p16Nuclei | Percentage (%) of myonuclei expressing ≥2 *p16* mRNA transcript foci | " |
| Percentage of fibre foci positive for ≥2 *p16* (%) | %2+p16Fibre | Percentage (%) of myofibre expressing ≥2 *p16* mRNA transcript foci | " |
| Percentage of any foci positive for ≥2 *p16* (%) | %2+p16Any | Percentage (%) of both myonuclei and myofibre expressing ≥2 *p16* mRNA transcript foci | " |
| Percentage of nuclear foci positive for *p16* (%) | %p16+Nuclei | Percentage (%) of myonuclei expressing any number of *p16* mRNA transcript foci (≥1) | " |
| Percentage of fibre foci positive for *p16* (%) | %p16+Fibre | Percentage (%) of myofibres expressing any number of *p16* mRNA transcript foci (≥1) | " |
| Percentage of any foci positive for *p16* (%) | %p16+Any | Percentage (%) of both myonuclei and myofibre expressing any number of *p16* mRNA transcript foci (≥1) | " |
| TAF and γH2A.X | | Telomeres are specialised structures the ends of chromosomes comprised of tandem 5'-TTAGGG-3' repeats that play a vital role in maintaining genomic stability [S4]. Telomere dysfunction results in the activation of a persistent DNA-damage response (DDR) which has been shown to trigger and maintain senescence in several tissues during ageing [S5]. γH2A.X, a phosphorylated histone variant H2A.X at serine 139 (Ser139), is the DDR protein that activates DNA-damage mediators [S6]. The colocalisation between telomeres and the DDR protein γH2A.X is otherwise known as TAF (telomere-associated DNA damage foci). | Immunofluorescence *in situ* hybridisation (immuno-FISH) |
| Percentage positive for ≥2 TAF positive (%) | %TAF2+ | Percentage (%) of myonuclei expressing ≥2 TAF signals | " |
| Percentage positive for ≥3 TAF positive (%) | %TAF3+ | Percentage (%) of myonuclei expressing ≥3 TAF signals | " |
| Percentage positive for γH2A.X (%) | % γH2A.X | Percentage (%) of myonuclei expressing % γH2A.X signal | Immunofluorescence |
| γH2A.X positive as a proportion of TAF positive (%) | TAF% γH2A.X | Percentage of myonuclei expressing % γH2A.X signal as a proportion of TAF positive nuclei | Immuno-FISH |

| | | | |
|---|---|---|---|
| HMGB1 | | HMGB1 (High Mobility Group Box 1) is a DNA binding non-histone protein that acts as an alarmin, signaling cellular damage when released extracellularly and creating an inflammatory response associated with the senescence-associated secretory phenotype (SASP). The loss of HMGB1 expression with age have been identified as a marker of senescence both *in vivo* and *in vitro* [S7]. | Immunofluorescence (IF) |
| Percentage of nuclei positive for HMGB1 (%) | %HMGB1+ | Percentage (%) of myonuclei expressing HMGB1 signal | “ |
| Lamin B1 | | Lamin B1 is a nuclear envelope protein involved in nuclear stability. The loss of Lamin B1 with age have been identified as a marker of cellular senescence [S8]. | Immunofluorescence |
| Percentage of nuclei positive for Lamin B1 (%) | %LaminB1+ | Percentage (%) of myonuclei positive for Labin B1 signal | “ |

**Supplementary Table 2.** Reagents and Assays

| Item | Supplier | Catalogue Number |
|---|---|---|
| 4% Paraformaldehyde in PBS | Santa-Cruz Biotechnology | sc-281692 |
| Avidin/Biotin Blocking Kit | Vector Laboratories | SP-2001 |
| Bovine Serum Albumin (BSA) | Sigma-Aldrich | A3059 |
| Cy-3-labelled telomere specific (CCCTAA) peptide nuclei acid (CCCTAA) peptide nuclei acid probe (594 nm) | Panagene | F1002-5 |
| Deionised Formamide | Amresco | 606 |
| EcoMount | Biocare Medical | EM897L |
| EDTA | Sigma-Aldrich | E9884 |
| Eosin Y | ScyTek Laboratories | EY0500 |
| Ethanol, Absolute | Fisher Scientific | 64-17-5 |
| Fluorescein Avidin DCS | Vector Laboratories | A2011 |
| Formamide | Sigma-Aldrich | F7508 |
| Haematoxylin | Sigma-Aldrich | MHS16 |
| Histo-Clear | National Diagnostics | HS-200 |
| Hydrogen Chloride | Sigma-Aldrich | HX0603 |
| Magnesium Chloride | BDH UK | 101494V |
| Malic Acid | Sigma-Aldrich | M0375 |
| Methanol, Absolute | Fisher Scientific | 67-56-1 |
| Normal Goat Serum | Vector Laboratories | S-1000 |
| Phosphate Buffered Saline, 10X | Sigma-Aldrich | D1408 |
| Picro Sirius Red Stain Kit | Abcam | ab150681 |
| ProLong Gold Mounting Media with DAPI | Thermo Fisher | 15260719 |
| RNAscope 2.5 HD Detection Reagent - RED | Advanced Cell Diagnostics | 322360 |
| RNAscope $H_2O_2$ & Protease Plus Reagents | Advanced Cell Diagnostics | 322330 |
| RNAscope Probe Hs-CDKN2A | Advanced Cell Diagnostics | 310181 |
| RNAscope Target Retrieval Reagents | Advanced Cell Diagnostics | 322000 |
| RNAscope Wash Buffer Reagents | Advanced Cell Diagnostics | 310091 |
| Roche Blocking reagent | Roche | 11096176001 |
| Sodium Chloride | Sigma-Aldrich | S7653 |
| Sodium Citrate | Sigma-Aldrich | W302600 |
| Tris | Sigma-Aldrich | T1503 |
| Wheat Germ Agglutinin (647nm) | Thermo Fisher Scientific | W11261 |

**Supplementary Table 3.** Stock solutions

| Solution | Components |
|---|---|
| 2x Saline Sodium Citrate (SSC) Buffer (pH 7.0) | 17.532g sodium chloride (0.3M)<br>8.823g sodium citrate (0.03M)<br>1L distilled water |
| 4% Paraformaldehyde | 40g paraformaldehyde<br>1L PBS |
| Citric Acid Buffer (0.1M, pH 6.0) | 29.41g of trisodium citrate<br>1L distilled water |
| Tris-EDTA Buffer (1M, pH 7.0) | 15.759g Tris (0.1M, pH 8.0)<br>2.92g EDTA (0.01M, pH 8.0)<br>1L distilled water |
| FISH Wash Buffer | 70ml formamide<br>30ml 2x SSC |
| ImmunoFISH Hybridisation Buffer | 2.5µl Tris (1M, pH 7.2)<br>21.4µl magnesium chloride buffer<br>175µl deionised formamide<br>1µl Cy-3-labelled telomere specific (CCCTAA) peptide nuclei acid probe<br>12.5µl blocking reagent<br>(2µl 10x blocking reagent (Roche) in 18µl malic acid (pH 7.5))<br>33.6µl distilled water |
| Magnesium Chloride Buffer (pH 7.0) | 0.119g magnesium chloride<br>86.445mg citric acid<br>0.582g hydrogen phosphate<br>50 ml distilled water<br>Maleic acid buffer<br>2.901g maleic acid<br>250ml deionised water |
| Malic Acid Buffer (pH 7.5) | 100mM malic acid<br>150mM NaCl in distilled water |
| Roche Blocking Reagent | 150mM NaCl in distilled water |

**Supplementary Table 4.** Antibodies[a] and dilutions

| Primary Antibodies | | | | | Secondary Antibodies | | Tertiary Antibodies/Development Kit | |
|---|---|---|---|---|---|---|---|---|
| Antigen | Supplier | | Species | Dilution | Antibody | Dilution | Antibody | Dilution |
| γH2A.X (S139) | Cell Signalling | 9718S | Rabbit | 1:250 | Goat anti-rabbit, biotinylated (BA-1000) | 1:200 | DCS Fluorescein (Vector Labs) | 1:500 |
| HMGB1 | Abcam | ab18526 | Rabbit | 1:500 | Goat anti-rabbit, Alexa Fluor 594 (ab150080) | 1:500 | | |
| Lamin B1 | Abcam | ab16048 | Rabbit | 1:500 | Goat anti-rabbit, Alexa Fluor 488 (ab150077) | 1:500 | | |

[a]All antibodies polyclonal unless otherwise stated.

**Supplementary Table 5.** Quantification of additional cellular senescence markers in skeletal muscle in men and women in the MASS_Lifecourse Study

| **Cellular senescence marker**[a] | **Abbreviation** | **Men** | **Women** | ***P* value**[b] |
|---|---|---|---|---|
| Percentage of nuclear foci positive for p16 (%) | %p16+Nuclei | 27.6 (17.8, 35.4) | 30 (23.8, 34.4) | 0.61 |
| Percentage of fibre foci positive for p16 (%) | %p16+Fibre | 22.5 (11.3, 28.0) | 12.9 (7.9, 19.0) | 0.09 |
| Percentage of any foci positive for p16 (%) | %p16+Any | 39.5 (35.7, 48.9) | 36.6 (33.0, 51.3) | 0.49 |
| Percentage positive for γH2A.X (%) | % γH2A.X | 54.0 (47.0, 65.5) | 52.0 (46.0, 58.5) | 0.39 |

[a]Values shown are median (interquartile range, IQR) of within participant value.
[b]Differences between men and women analysed using Wilcoxon rank-sum tests.

# Appendix 3

## Supplementary Figures and Figure Legends

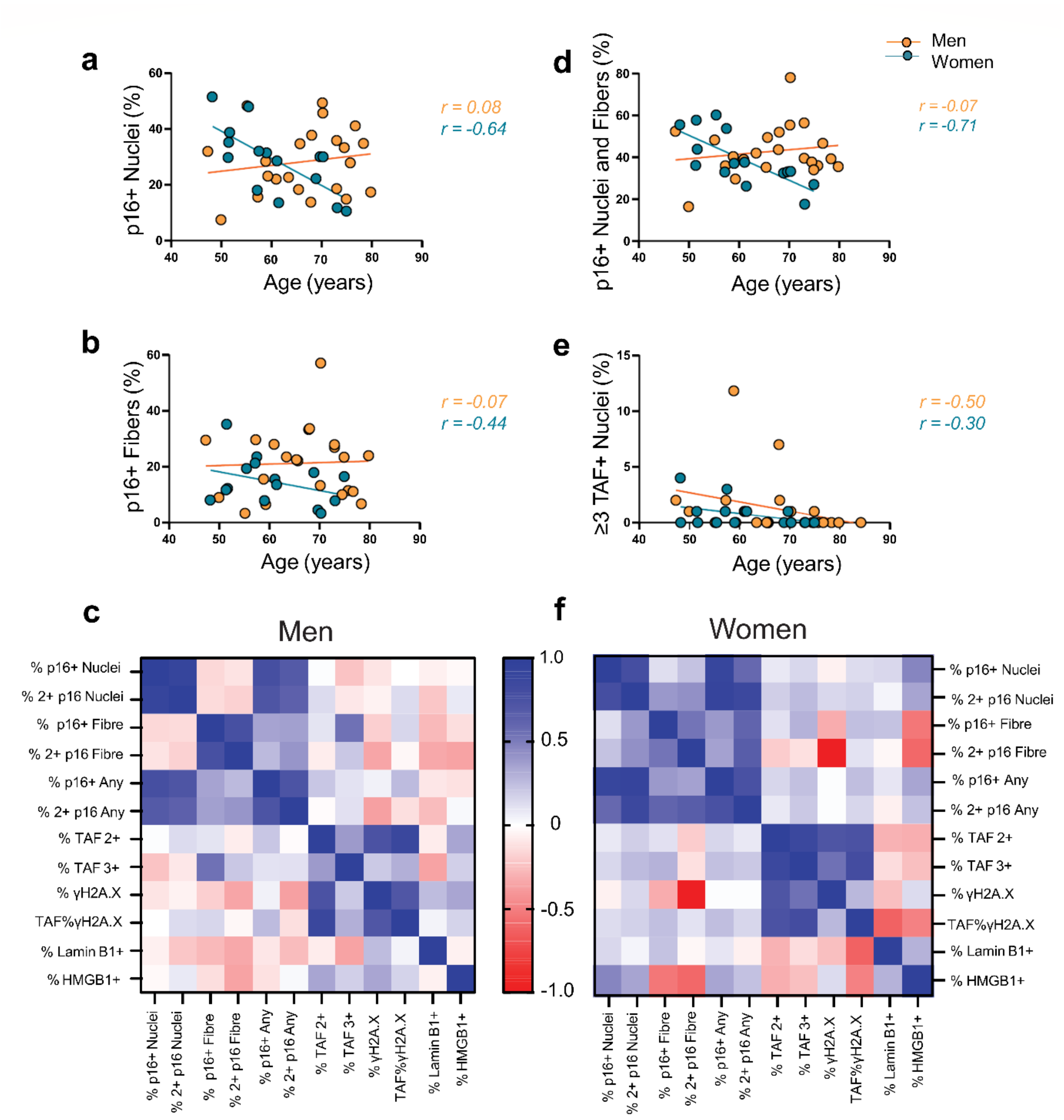


**Supplementary Figure 1**. Changes in cellular senescence marker expression in skeletal muscle with age and associations between senescence markers in men and women in the MASS_Lifecourse Study.

Graphs portraying changes in (**a**) percentage *p16* positive nuclei, (**b**) percentage *p16* positive fibers, (**d**) percentage *p16* positive fibers and nuclei, and (**e**) percentage nuclei positive for at least 3 TAF. Heatmaps portraying correlation coefficients between senescence markers in middle-aged and old (**c**) men and (**f**) women in the

MASS_Lifecourse Study. Correlations were examined using Spearman's correlation test. Regression lines are presented in orange for men, and in teal for women. Graphs and heatmaps were generated in Prism 9.0.

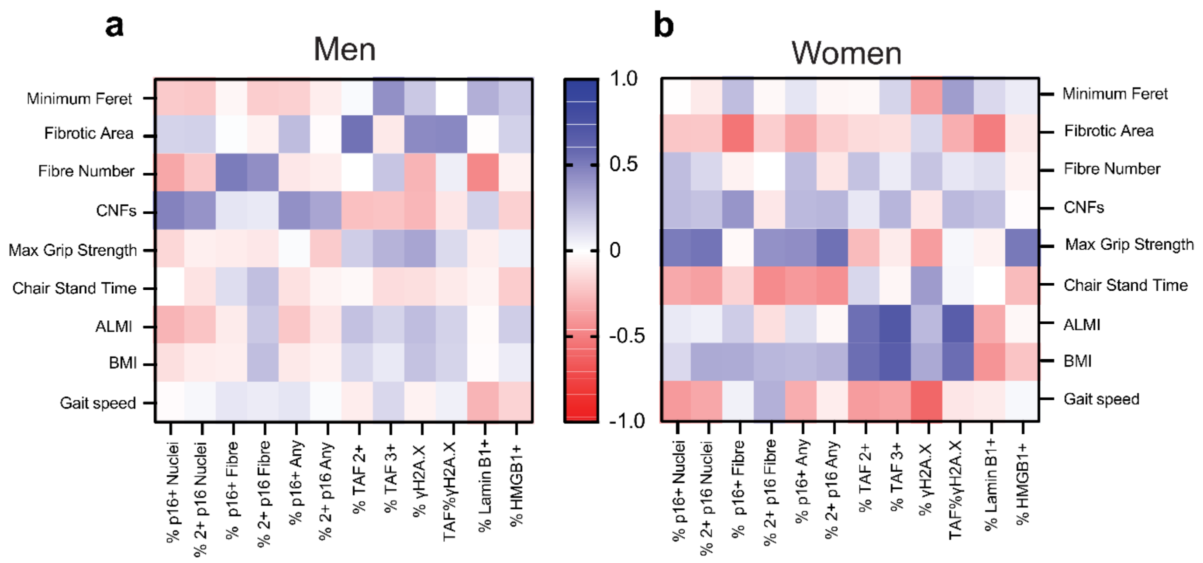


**Supplementary Figure 2.** Associations between markers of cellular senescence, morphological characteristic, and physical function with age in men and women in the MASS_Lifecourse Study

Heatmaps portraying correlation coefficients between senescence markers, muscle function, and indicators of muscle ageing in middle-aged and old (**a**) men and (**b**) women in the MASS_Lifecourse Study. Correlations were determined using Spearman's correlation test. Heatmaps were generated in Prism 9.0.

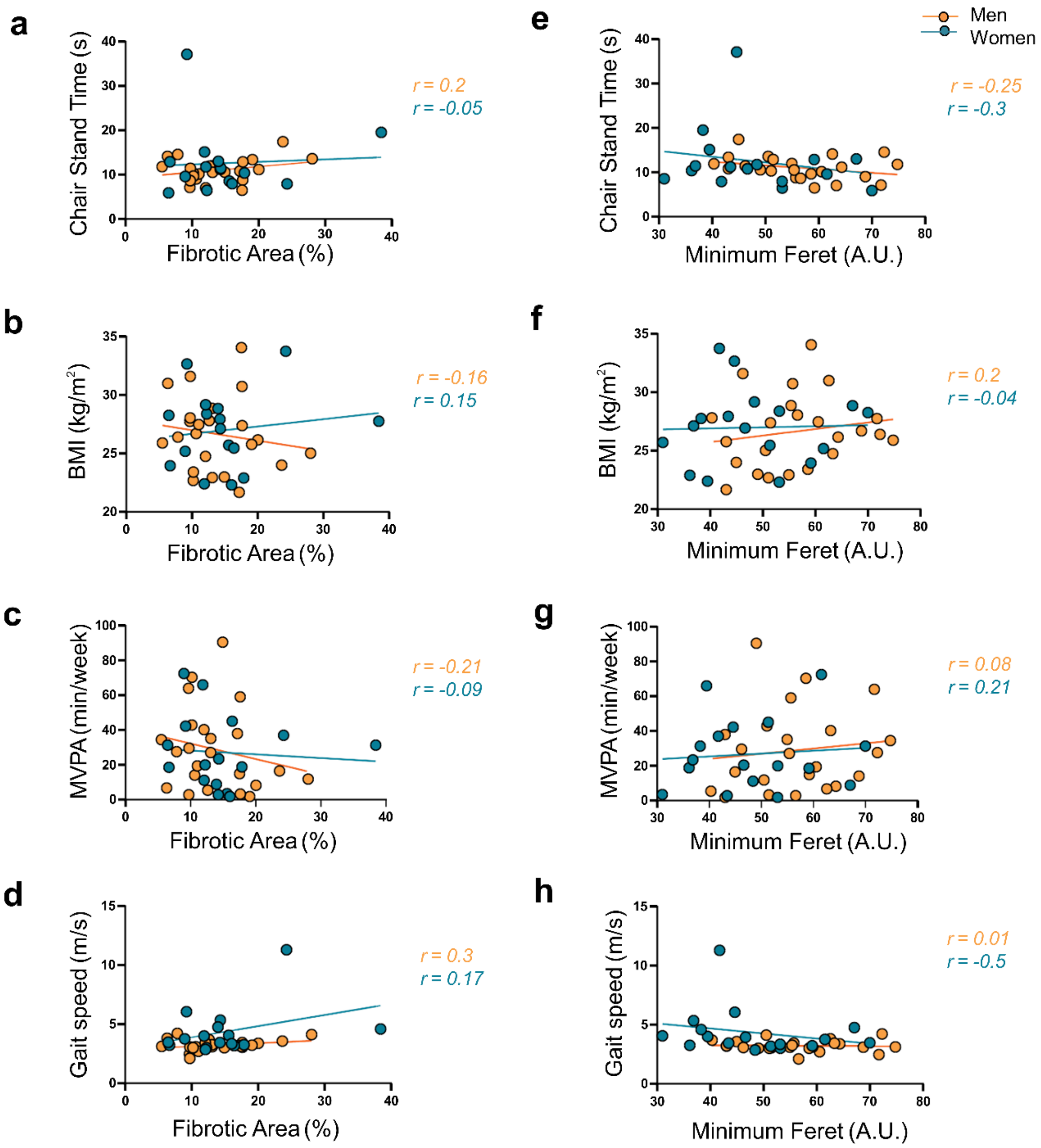


**Supplementary Figure 3.** Associations between morphological characteristics and physical function with age in men and women in the MASS_Lifecourse Study.

Graphs portraying correlations between percentage fibrotic area and (**a**) 5-chair stand time, (**b**) body mass index (BMI), (**c**) moderate to vigorous physical activity (MVPA; daily minutes), and (**d**) gait speed (meters per second). Graphs portraying correlations between minimum feret (fibre size) and (**e**) 5-chair stand time, (**f**) body mass index (BMI), (**g**) moderate to vigorous physical activity (MVPA; daily minutes), and (**h**) gait speed (meters per second). Correlations were determined using Spearman's correlation test. Graphs were generated in Prism 9.0.

## Supplementary References

S1. Van Hees VT, Fang Z, Zhao JH, Heywood J, Mirkes E, Sabia S, Migueles J. GGIR: Raw accelerometer data analysis. R Packag version. 2019:18–1.

S2. Coppé JP, Rodier F, Patil CK, Freund A, Desprez PY, Campisi J. Tumor suppressor and aging biomarker p16(INK4a) induces cellular senescence without the associated inflammatory secretory phenotype. J Biol Chem. 2011; 286:36396–36403.

S3. Campisi J. Cellular senescence as a tumor-suppressor mechanism. Trends Cell Biol. 2001;11:S27–31.

S4. Blackburn EH. Telomeres. Trends Biochem Sci. 1991;16:378–381.

S5. Hewitt G, Jurk D, Marques FD, Correia-Melo C, *et al*. Telomeres are favoured targets of a persistent DNA damage response in ageing and stress-induced senescence. Nat Commun. 2012;3:708.

S6. Rogakou EP, Pilch DR, Orr AH, Ivanova VS, Bonner WM. DNA double-stranded breaks induce histone H2AX phosphorylation on serine 139. J Biol Chem. 1998; 273:5858–5868.

S7. Davalos AR, Kawahara M, Malhotra GK, *et al*. p53-dependent release of Alarmin HMGB1 is a central mediator of senescent phenotypes. J Cell Biol. 2013; 201:613–629.

S8. Freund A, Laberge RM, Demaria M, Campisi J. Lamin B1 loss is a senescence-associated biomarker. Mol Biol Cell. 2012;23:2066–2075.
